# Supplementary material for: FlowMol3: Flow Matching for 3D De Novo Small-Molecule Generation
Source: ArXiv. 2025 Aug 18:arXiv:2508.12629v1. Preprint. [Version 1] (PMC12393239)
Supplement: Supplement 1 [file NIHPP2508.12629v1-supplement-1.pdf]

FlowMol3 Supplementary Information

Contents

S1 Additional Data on PoseBusters Validity **1**

S2 Example Molecules **3**

S3 The Coupling Distribution **3**

S4 Training Details and Hyperparameters **4**

S5 Additional Techniques for DFM Sampling **5**
- S5.1 Remasking via Stochasticity 5
- S5.2 Low-Temperature Sampling 5
- S5.3 Purity Sampling 6

S6 GVP with Cross Product **6**

S7 Examples of Out-of-Distribution Ring Systems **7**

S8 Individual Functional Group Frequencies **9**

S1 Additional Data on PoseBusters Validity

In the main paper we report the percent of molecules that are valid as determined by the PoseBusters suite[1] under the name "% PB-Valid". A model is considered PB-valid if it passes all of a series of tests. Here we present the pass-rate for each individual test run by PoseBusters; these results are shown in Table S1.

Table S1: Pass rates on individual PoseBusters tests with 95% confidence intervals

	FlowMol3	SemlaFlow	Megalodon	ADiT
mol pred loaded	100.0 ± 0.0	100.0 ± 0.0	100.0 ± 0.0	100.0 ± 0.0
sanitization	99.9 ± 0.1	95.4 ± 0.5	94.7 ± 0.2	99.9 ± 0.0
inchi convertible	99.9 ± 0.1	95.4 ± 0.5	94.7 ± 0.3	99.9 ± 0.0
all atoms connected	98.9 ± 0.2	97.3 ± 1.1	97.8 ± 0.5	94.6 ± 0.5
bond lengths	100.0 ± 0.0	99.6 ± 0.2	99.9 ± 0.1	96.0 ± 0.3
bond angles	100.0 ± 0.0	100.0 ± 0.0	100.0 ± 0.0	95.4 ± 0.6
internal steric clash	94.1 ± 0.5	96.7 ± 0.6	94.4 ± 1.1	92.2 ± 0.4
aromatic ring flatness	100.0 ± 0.0	100.0 ± 0.0	100.0 ± 0.0	100.0 ± 0.0
non-aromatic ring non-flatness	99.6 ± 0.1	99.7 ± 0.1	99.8 ± 0.2	97.3 ± 0.3
double bond flatness	99.3 ± 0.3	99.2 ± 0.2	99.4 ± 0.2	99.9 ± 0.1
internal energy	100.0 ± 0.0	99.9 ± 0.1	99.9 ± 0.1	94.4 ± 0.3
all	91.9 ± 0.7	88.5 ± 1.3	86.6 ± 0.7	82.7 ± 0.8

S2 Example Molecules

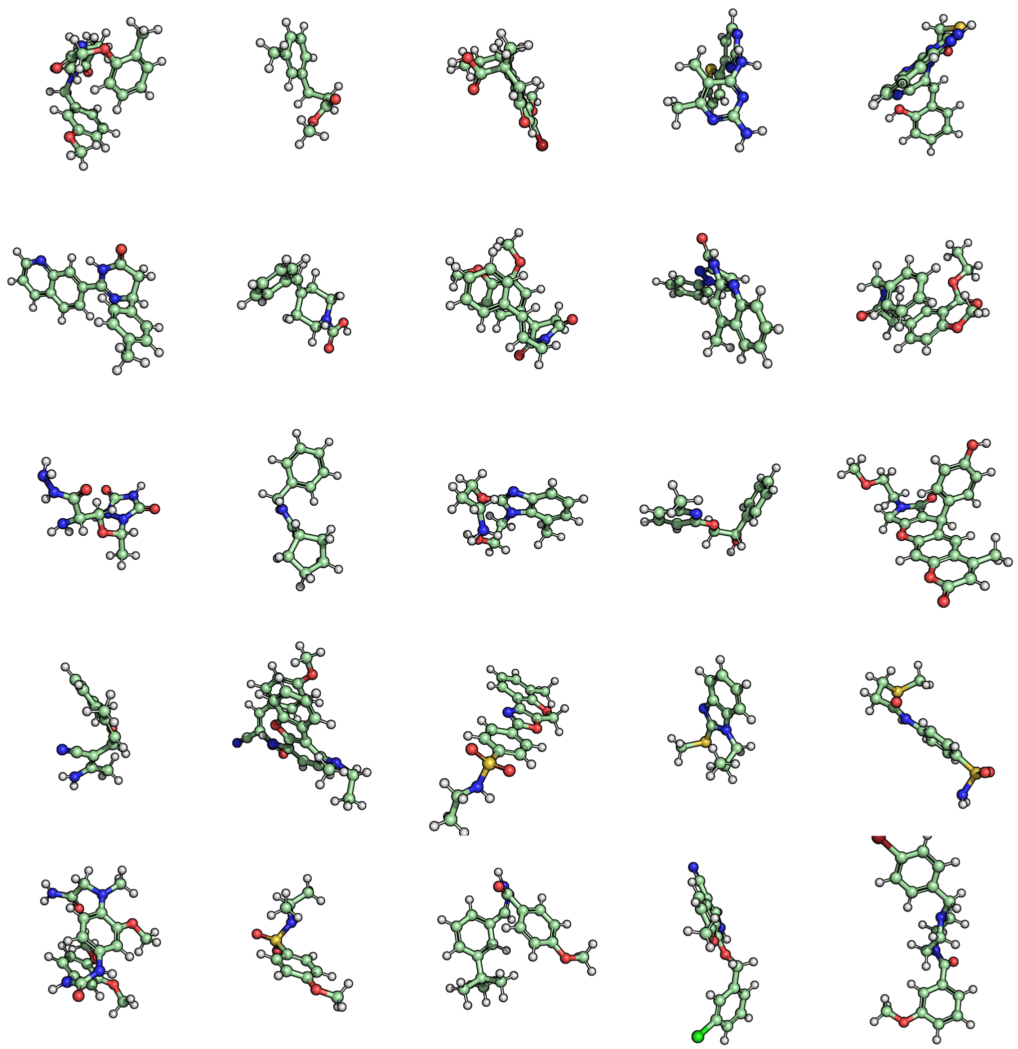

Figure S1: Example molecules sampled from FlowMol3

S3 The Coupling Distribution

Since we chose the conditioning variable for our conditional probability paths to be pairs of initial and final molecules (g_0, g_1), we must also define a method

of obtaining such paired samples of initial and final molecules. In other words, we must define the distribution from which our conditioning variable is obtained $p(g_0, g_1)$. This is generally referred to as a coupling distribution[2–4].

The simplest choice of the coupling distribution would be an independent coupling:

$$p(g_0, g_1) = p(g_0)p(g_1) \tag{1}$$

In FlowMol, the coupling distribution factorizes over modalities.

$$p(g_0, g_1) = p(X_0, X_1)p(A_0, A_1)p(C_0, C_1)p(E_0, E_1) \tag{2}$$

All discrete modalities are given the independent coupling:

$$p(A_0, A_1) = p(A_0)p(A_1) \tag{3}$$

That is, the the target value (A_1) is obtained from the dataset, and the corresponding prior value (A_0) is always a sequence of mask tokens, independent of the target value.

However, in continuous flow matching, the independent coupling can introduce pathologies that impair model performance such as having a large transport cost (distances between prior and target samples), and intersecting conditional paths. Several works identify this pathology and propose various forms of coupling distributions that involve drawing independent samples and then aligning them in some fashion[3,5,6].

For continuous modalities, we implement a technique similar to the Equivariant Optimal Transport Coupling proposed by Klein *et al.*[6]. We first obtain the $t = 0$ atom coordinates as independent samples from a standard Gaussian $X_0 \sim \prod_{i=1}^{N} \mathcal{N}(x_0^i | 0, \mathbb{I}_3)$. We then align the prior coordinates X_0 to the ground-truth coordinates X_1 via the Kabsch algorithm. Next, we permute the order of atoms in X_0. This can be thought of as permuting the intial positions of each atom. We specifically choose the permutation that minimizes the sum of distances between initial and final positions across all atoms (this is known as solving the assignment problem).

S4 Training Details and Hyperparameters

FlowMol3 is trained with 6 Molecule Update Blocks. Atoms contain 256 hidden scalar features and 32 hidden vector features. Edges contain 128

hidden features. All models are trained for 20 epochs. GEOM models are trained on 4xL40 GPUs with an adaptive batch-size that typically yields 15-25 graphs per batch per GPU. Training takes approximately 4-5 days.

As described in Section 2.4 of the main paper, the overall loss for training FlowMol3 is a weighted sum of per-modality flow matching losses. The loss weights $(\lambda_X, \lambda_A, \lambda_C, \lambda_E)$ are set to (3, 0.4, 1, 2).

All model hyperparameters are visible in the config files provided in our github repository.

S5 Additional Techniques for DFM Sampling

Here we discuss three techniques we apply to sampling discrete modalities in FlowMol3: remasking, low-temperature sampling, and purity sampling.

S5.1 Remasking via Stochasticity

Recall our marginal probability velocity ((12) from the main paper, reproduced here)

$$u^i(j, A_t) = \frac{1+\eta t}{1-t} p^{\theta}_{1|t}(a^i_1 = j|A_t)\delta_M(a^i_t) + \eta(1-\delta_M(a^i_t))\delta_M(j) \tag{4}$$

Includes the hyperparameter $\eta \geq 0$ which can be chosen at inference time. η can be called the "stochasticity parameter". If we set $\eta = 0$ then once a sequence element (i.e., an atom type or a bond order) is unmasked, that value is fixed for the remainder of the trajectory. If we choose $\eta > 0$, sequence elements may be remasked and unmasked again repeatedly throughout a trajectory; enabling the denoiser to correct or change past decisions. We find that enabling remasking significantly enhances sample quality. All FlowMol results obtained in this paper are done so using $\eta = 30$.

S5.2 Low-Temperature Sampling

Recall that for discrete modalities our neural networks directly approximate the distribution of states at $t = 1$ for each sequence element. In the context of atom types, for example, this quantity is denoted $p^{\theta}_{1|t}(a^i_1|A_t)$.

We re-normalize logits obtained from the model using a fixed temperature τ:

$$p^{\theta}_{1|t}(a^i_1|A_t) = \text{softmax}\left(\tau^{-1}\log p^{\theta}_{1|t}(a^i_1|A_t)\right) \quad (5)$$

We find that low-temperature sampling, which biases the sampled discrete states towards the most confident ones, to be critical for model performance. In practice we use $\tau = 0.05$.

S5.3 Purity Sampling

Under the base DFM formulation, at each integration step, the probability of unmasking each currently-masked sequence element is $\Delta t\frac{1+\eta t}{1-t}$. Every masked token has an equal probability of getting unmasked.

Rather than doing this, we implement purity sampling as described in Campbell *et al.*[7]. We select which currently-masked elements to unmask using a proxy for model confidence. The proxy used for model confidence is the "purity": the maximum category probability output by the model.

During inference, at each integration step, we first sample the number of elements to unmask from a binomial distribution with n equal to the number of mask tokens in the sequence and $p = \Delta t\frac{1+\eta t}{1-t}$. Then after we obtain $k \sim \text{Binomial}(n, p)$, we select the top-$k$ sequence elements with the highest "purity"; these are the tokens that will be unmasked.

S6 GVP with Cross Product

A geometric vector perception (GVP) can be thought of as a single-layer neural network that applies linear and point-wise non-linear transformation to its inputs. The difference between a GVP and a conventional feed-forward neural network is that GVPs operate on two distinct data types: scalars and vectors. GVPs also allow these data types to exchange information while preserving equivariance of the output vectors. The original GVP only applied linear transformations to the vector features and as a result produces output vectors that are E(3)-equivariant.

We introduce a modification to the GVP as its presented in Jing *et al.*[8]; specifically we perform a cross product operation on the input vectors. The motivation for this is that the cross product is *not* equivariant to reflections. We refer the reader to Appendix F of Schneuing *et al.*[9] for a detailed discussion of the equivariance of cross products. As a result, the version of GVP we present here is SE(3) equivariant. The benefit of being SE(3) equivariant

rather than E(3) equivariant is that the generative model becomes sensitive to chiral centers in molecules, since reflecting the molecule will produce different model outputs. The operations for our cross product enhanced GVP are described in Algorithm 1.

Algorithm 1 Geometric Vector Perceptron with Cross Product

Input: Scalar and vector features: $(s, v) \in \mathbb{R}^f \times \mathbb{R}^{\nu \times 3}$
Output: Scalar and vector features: $(s', v') \in \mathbb{R}^j \times \mathbb{R}^{\mu \times 3}$
Hyperparameter: Number of hidden vector features $n_h \in \mathbb{Z}^+$
Hyperparameter: Number of cross product features $n_{cp} \in \mathbb{Z}^+$
$v_h \leftarrow W_h v \quad \in \mathbb{R}^{n_h \times 3}$
$v_{cp} \leftarrow W_{cp} v \quad \in \mathbb{R}^{2n_{cp} \times 3}$
$v_{cp} \leftarrow v_{cp}[: n_{cp}] \times v_{cp}[n_{cp} :] \quad \in \mathbb{R}^{n_{cp} \times 3}$ // cross product
$v_{h+cp} \leftarrow \text{Concat}(v_h, v_{cp}) \quad \in \mathbb{R}^{(n_h + n_{cp}) \times 3}$
$v_\mu \leftarrow W_\mu v_{h+cp} \quad \in \mathbb{R}^{\mu \times 3}$
$s_{h+cp} \leftarrow \|v_{h+cp}\| \quad \in \mathbb{R}^{n_h + n_{cp}}$
$s_{f+h+cp} \leftarrow \text{Concat}(s, s_{h+cp})$’
$s_j \leftarrow W_j s_{f+h+cp} + b_j \quad \in \mathbb{R}^j$
$s' \leftarrow \sigma(s_j) \quad \in \mathbb{R}^j$
$v' \leftarrow \sigma_g \left(W_g[\sigma^+(s_m)] + b_g\right) \odot v_\mu$ (row-wise) $\quad \in \mathbb{R}^{\mu \times 3}$
return (s', v')

S7 Examples of Out-of-Distribution Ring Systems

To provide intuition about the purpose or significance of the OOD ring system metric, we provide examples of OOD ring systems produced by evaluated models in Figure S2.

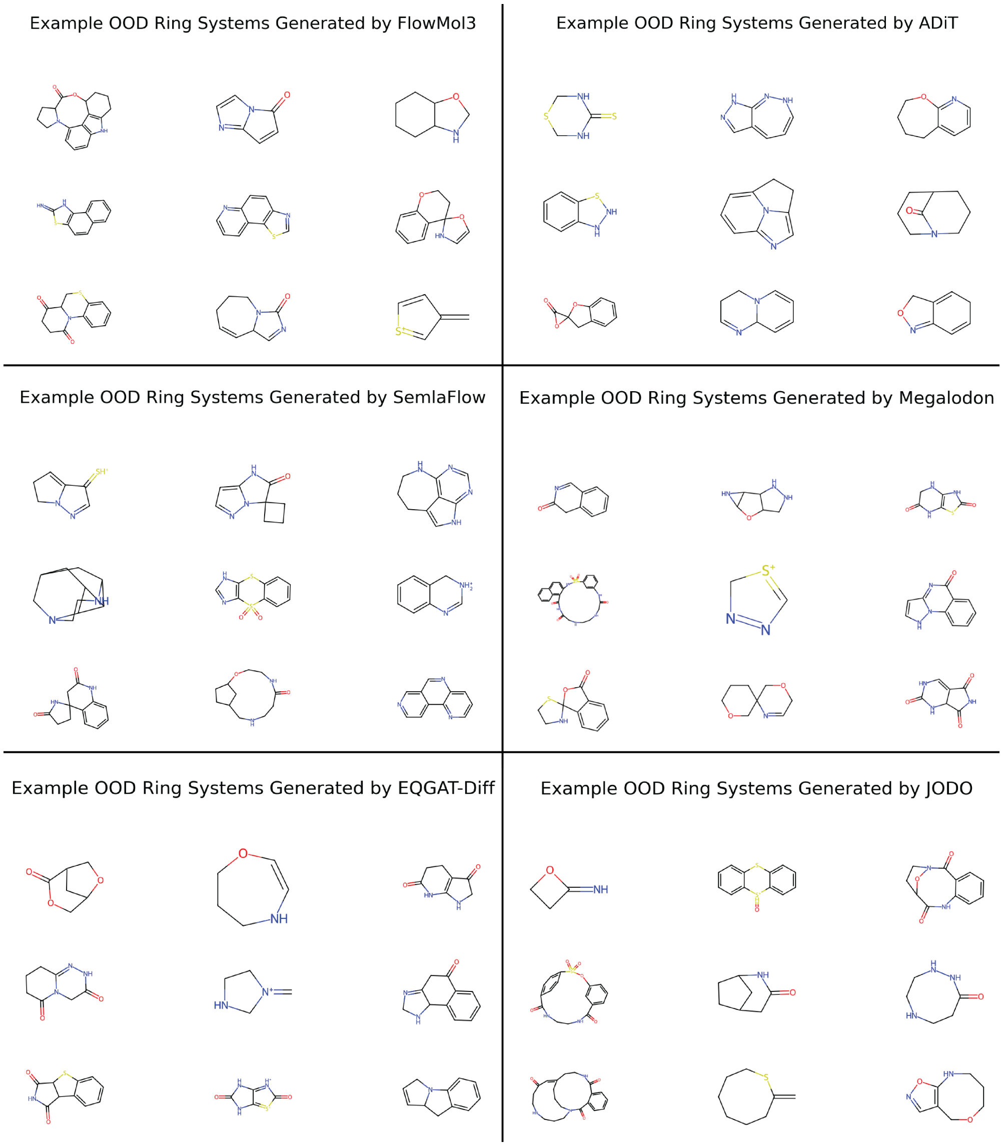

Figure S2: Examples of OOD ring systems (ring systems that do not appear in ChEMBL) that were produced by several of the models evalauted in this paper. The specific OOD ring systems were selected randomly from the set of all OOD ring systems found.

S8 Individual Functional Group Frequencies

We show the functional group frequencies for the 36 most commonly occuring functional groups evaluated in the following figures. The y-axis in all figures is the frequency of the functional group per 100 sampled molecules.

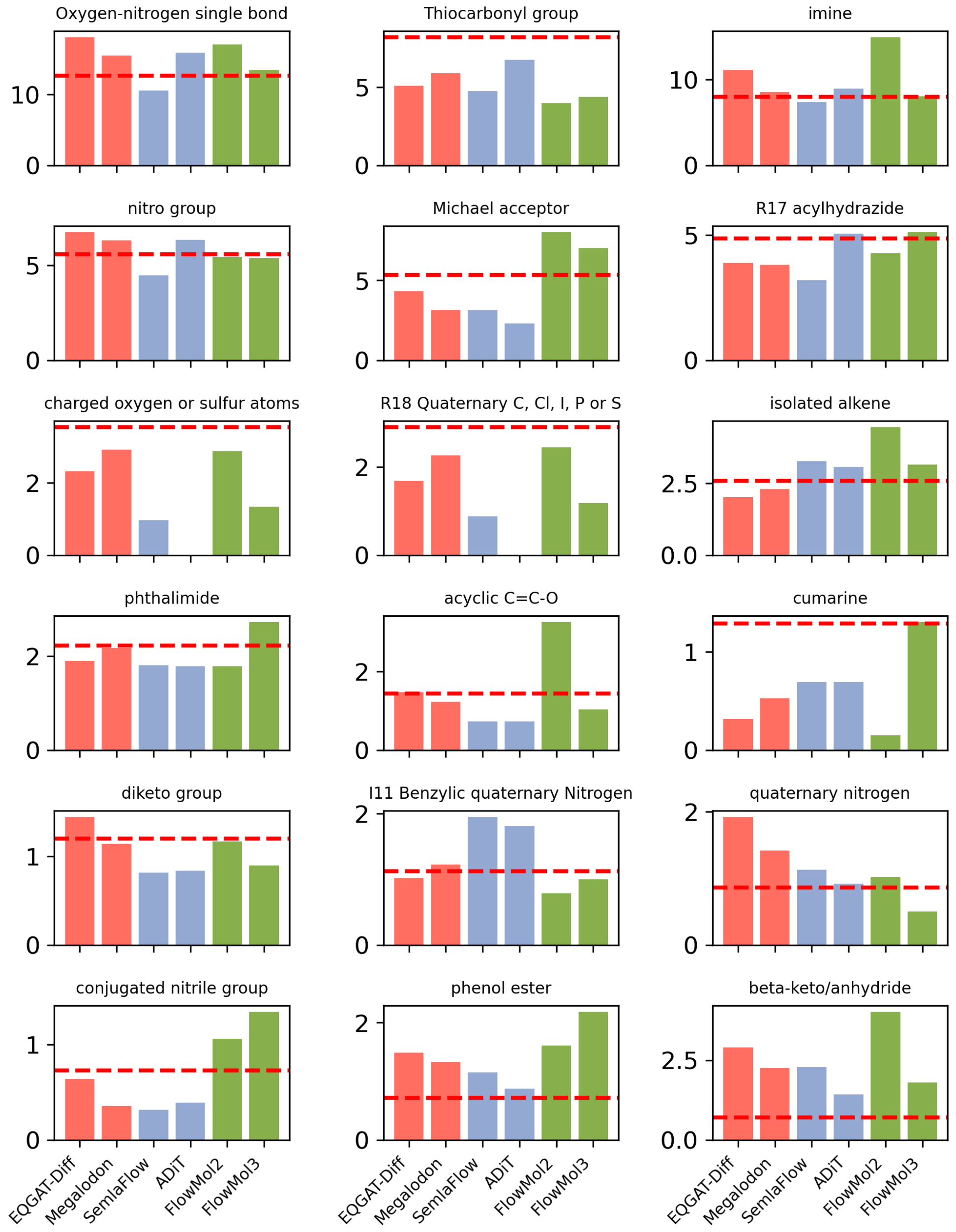
Oxygen-nitrogen single bond
Thiocarbonyl group
imine
nitro group
Michael acceptor
R17 acylhydrazide
charged oxygen or sulfur atoms
R18 Quaternary C, Cl, I, P or S
isolated alkene
phthalimide
acyclic C=C-O
cumarine
diketo group
I11 Benzylic quaternary Nitrogen
quaternary nitrogen
conjugated nitrile group
phenol ester
beta-keto/anhydride
EQGAT-Diff
Megalodon
SemlaFlow
ADiT
FlowMol2
FlowMol3

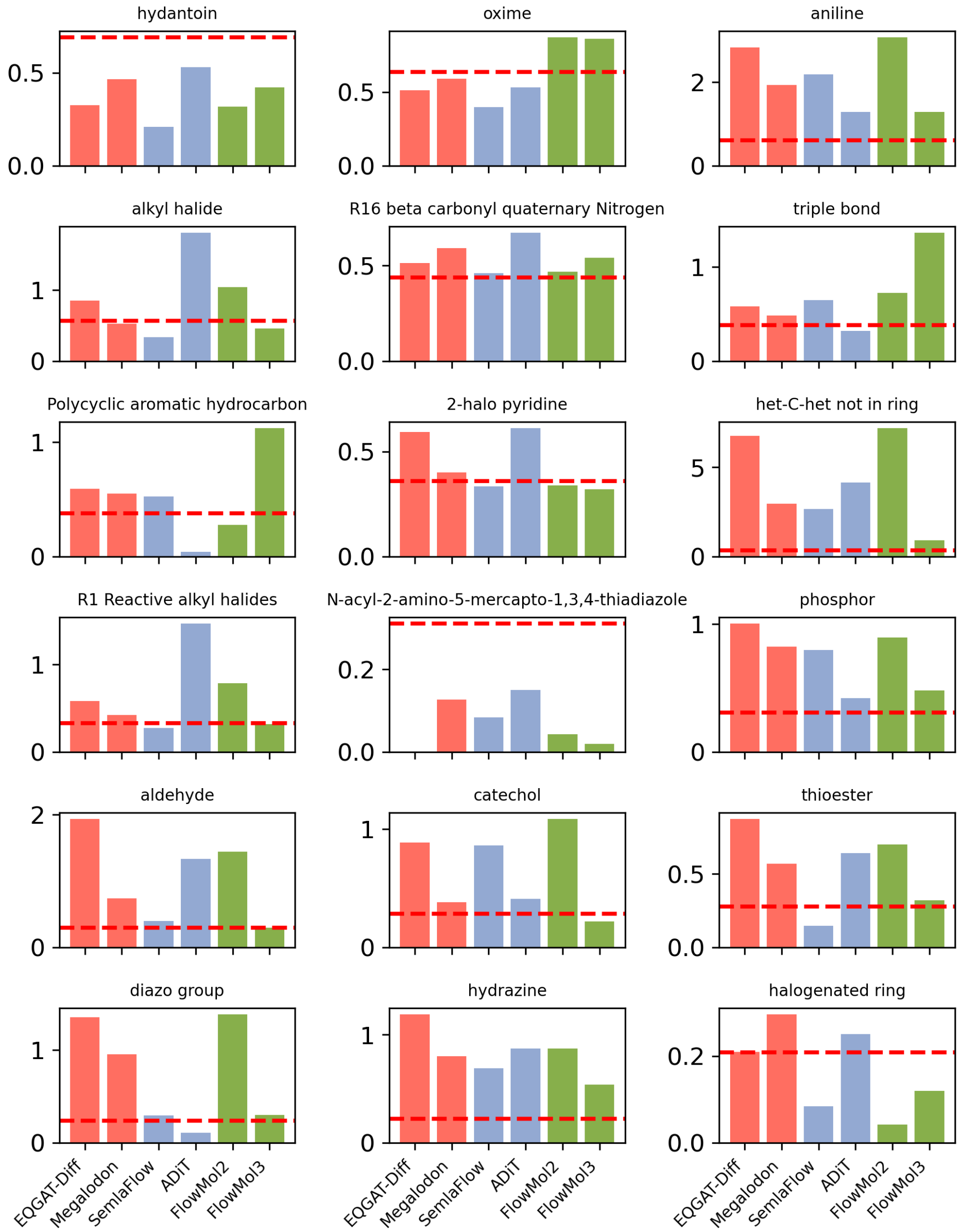
hydantoin
oxime
aniline
alkyl halide
R16 beta carbonyl quaternary Nitrogen
triple bond
Polycyclic aromatic hydrocarbon
2-halo pyridine
het-C-het not in ring
R1 Reactive alkyl halides
N-acyl-2-amino-5-mercapto-1,3,4-thiadiazole
phosphor
aldehyde
catechol
thioester
diazo group
hydrazine
halogenated ring
EQGAT-Diff
Megalodon
SemlaFlow
ADiT
FlowMol2
FlowMol3

References

[1] M. Buttenschoen, G. M. Morris and C. M. Deane, *Chemical Science*, 2024, **15**, 3130–3139.

[2] M. S. Albergo and E. Vanden-Eijnden, *Building Normalizing Flows with Stochastic Interpolants*, 2023, `http://arxiv.org/abs/2209.15571`, arXiv:2209.15571 [cs, stat].

[3] A. Tong, N. Malkin, G. Huguet, Y. Zhang, J. Rector-Brooks, K. Fatras, G. Wolf and Y. Bengio, *Improving and generalizing flow-based generative models with minibatch optimal transport*, 2023, `http://arxiv.org/abs/2302.00482`, arXiv:2302.00482 [cs].

[4] I. Gat, T. Remez, N. Shaul, F. Kreuk, R. T. Q. Chen, G. Synnaeve, Y. Adi and Y. Lipman, *Discrete Flow Matching*, 2024, `http://arxiv.org/abs/2407.15595`, arXiv:2407.15595 [cs].

[5] X. Liu, C. Gong and Q. Liu, *Flow Straight and Fast: Learning to Generate and Transfer Data with Rectified Flow*, 2022, `http://arxiv.org/abs/2209.03003`, arXiv:2209.03003 [cs].

[6] L. Klein, A. Krämer and F. Noé, *Equivariant flow matching*, 2023, `http://arxiv.org/abs/2306.15030`, arXiv:2306.15030 [physics, stat].

[7] A. Campbell, J. Yim, R. Barzilay, T. Rainforth and T. Jaakkola, *Generative Flows on Discrete State-Spaces: Enabling Multimodal Flows with Applications to Protein Co-Design*, 2024, `http://arxiv.org/abs/2402.04997`, arXiv:2402.04997 [cs, q-bio, stat].

[8] B. Jing, S. Eismann, P. N. Soni and R. O. Dror, *Equivariant Graph Neural Networks for 3D Macromolecular Structure*, 2021, `http://arxiv.org/abs/2106.03843`, arXiv:2106.03843 [cs, q-bio].

[9] A. Schneuing, Y. Du, C. Harris, A. Jamasb, I. Igashov, W. Du, T. Blundell, P. Lió, C. Gomes, M. Welling, M. Bronstein and B. Correia, *Structure-based Drug Design with Equivariant Diffusion Models*, 2023, `http://arxiv.org/abs/2210.13695`, arXiv:2210.13695 [cs, q-bio].
